# Open Versus Arthroscopic Reduction for Perilunate and Lunate Dislocations: A Systematic Review and Network Meta-Analysis

**DOI:** 10.1016/j.jhsg.2026.101010

**Published:** 2026-08-07

**Authors:** Ahmed Khaled Almarri, Yazan J. Alalwani, Mohammed H. Almuhideb, Leen Albraik, Layan Albraik, Raghad Yaser Sonbul, Mohammed Saud Aldofayan, Muteb N. Alotaibi, Lames S. Almohamadi, Wiam M. Alhoshani, Rahaf Alruwaili, Rana Ali Hassan Arab, Ahmed Y. Azzam, Bayan A. Ghalimah

**Affiliations:** ∗ Department of Orthopedic Surgery, College of Medicine, Imam Abdulrahman bin Faisal University, Dammam, Saudi Arabia; † College of Medicine, Imam Abdulrahman bin Faisal University, Dammam, Saudi Arabia; ‡ College of Medicine, Alfaisal University, Riyadh, Saudi Arabia; § College of Medicine, Imam Mohammad Ibn Saud Islamic University, Riyadh, Saudi Arabia; ‖ Department of Orthopaedics, King Faisal Specialist Hospital & Research Center, Riyadh, Saudi Arabia; ∗∗ College of Medicine, King Saud Bin Abdulaziz University for Health Sciences, Saudi Arabia; †† College of Medicine, Qassim University, Saudi Arabia; ‡‡ College of Medicine, Jouf University, Saudi Arabia; §§ Allith Hospital, Jeddah 1st Health Cluster, Jeddah, Saudi Arabia; ‖‖ Division of Global Health and Public Health, School of Nursing, Midwifery and Public Health, University of Suffolk, Ipswich, UK; ∗∗∗ Orthopedic Surgery Department, Faculty of Medicine, King Abdulaziz University, Rabigh, Kingdom of Saudi Arabia

**Keywords:** Arthroscopic reduction, Lunate, Open fixation, Perilunate

## Abstract

**Purpose:**

Perilunate and lunate dislocations represent complex wrist injuries requiring urgent reduction and stabilization. While open reduction has been the gold standard, arthroscopic techniques have emerged as less invasive alternatives. However, comparative evidence remains limited, and the management strategies are controversial.

**Methods:**

We conducted a systematic review and network meta-analysis following Preferred Reporting Items for Systematic Reviews and Meta-Analyses 2020 guidelines. Dedicated searches of major databases were performed up to June 16, 2025. Studies comparing open and arthroscopic reduction techniques for perilunate/lunate dislocations were included. Primary outcomes included functional scores (Mayo Wrist Score, Disabilities of the Arm, Shoulder, and Hand), whereas secondary outcomes encompassed complications, anatomical success, and grip strength recovery. Network meta-analysis was performed using random-effects models, with Grading of Recommendations Assessment, Development, and Evaluation framework for evidence quality assessment.

**Results:**

Thirty-four studies with total of 962 patients were included (open reduction: 584 patients, arthroscopic: 378 patients). Direct comparative evidence was limited to three studies (48 patients). Arthroscopic reduction demonstrated superior functional outcomes. Complication rates showed advantages for arthroscopic approaches. Subgroup analyses revealed surgeon experience significantly affected outcomes, with experienced surgeons achieving superior results and fewer complications. Greater arc fractures demonstrated higher complication rates regardless of approach.

**Conclusions:**

Arthroscopic reduction may offer functional advantages over open techniques for perilunate dislocations, especially when performed by experienced surgeons for acute, less complex injuries. However, evidence quality remains low, warranting high-quality randomized trials.

**Type of study/level of evidence:**

Therapeutic III.

Perilunate and lunate dislocations represent some of the most severe and complex injuries affecting the wrist, accounting for around 10% of all carpal injuries and constituting true orthopedic emergencies requiring immediate recognition and treatment.^1^, ^2^, ^3^

The complexity of these injuries extends beyond the immediate osseous and ligamentous disruption, as they frequently present with associated fractures of the scaphoid, capitate, hamate, or triquetrum, creating what are termed "greater arc" injuries when fractures are present or "lesser arc" injuries when purely ligamentous.^4^, ^5^, ^6^

Open reduction and internal fixation procedure has been considered the gold standard surgical strategy, allowing direct visualization of the injured structures, precise reduction of displaced bone fragments, and secure ligamentous repair or reconstruction. The open approach includes extensive soft tissue dissection through dorsal and often combined palmar approaches, providing good access to all injured structures but could increase the risk in resulting in significant surgical morbidity, prolonged recovery times, and risks of soft tissue complications including adhesion formation and stiffness.^7^, ^8^, ^9^

The advancements of arthroscopic techniques in wrist surgery have introduced the possibility of achieving similar anatomical restoration through minimally invasive approaches. Arthroscopic reduction offers advantages including preservation of soft tissue vascularity, reduced surgical trauma, faster recovery, and superior visualization of intra-articular structures. However, arthroscopic management of perilunate dislocations remains technically difficult, requiring higher expertise and equipment, and may not be suitable for all injury patterns or scenarios.^10^^,^^11^

Despite decades of experience with both approaches, the best dedicated and personalized management strategy for perilunate and lunate dislocations remains controversial. Individual studies have reported varying outcomes with both techniques, but the comparative evidence has been limited by the relative rarity of these injuries, heterogeneity in injury patterns, differences in surgical expertise, and inconsistency in outcome reporting. Previous studies have been limited by small sample sizes, lack of direct comparative studies, and methodological limitations that preclude definitive recommendations.^5^^,^^9^^,^^12^, ^13^, ^14^

Treatment decisions significantly impact outcomes, health-care utilization, and long-term function in typically young, active patients for whom wrist restoration is essential for occupational and recreational activities. The choice between approaches affects surgical risks, functional outcomes, complication rates, and revision likelihood.

We conducted a systematic review and network meta-analysis synthesizing evidence comparing open and arthroscopic reduction for perilunate and lunate dislocations, evaluating comparative effectiveness for functional outcomes, complications, and safety profiles while identifying factors influencing treatment selection.

## Methods

### Study design and search strategy

This systematic review and network meta-analysis was conducted in accordance with the Preferred Reporting Items for Systematic Reviews and Meta-Analyses 2020 guidelines and the extension statement for network meta-analyses.^15^^,^^16^

A search strategy was developed and implemented across multiple electronic databases from inception up to June 16, 2025. The databases searched included MEDLINE (via PubMed), Google Scholar, Cochrane Central Register of Controlled Trials (CENTRAL), Web of Science, and Scopus. The search strategy combined Medical Subject Headings terms and free-text keywords related to perilunate dislocations, lunate dislocations, arthroscopic reduction, open reduction, and carpal instability.

The search strategy included the following key terms and combinations: ("perilunate dislocation" OR "lunate dislocation" OR "perilunate instability" OR "lunate instability" OR "carpal dislocation" OR "transcaphoid perilunate" OR "trans-scaphoid perilunate" OR "greater arc injury" OR "lesser arc injury") AND ("arthroscopic reduction" OR "arthroscopic repair" OR "arthroscopic treatment" OR "arthroscopic fixation" OR "wrist arthroscopy" OR "carpal arthroscopy") AND ("open reduction" OR "open repair" OR "open treatment" OR "open fixation" OR "surgical treatment" OR "operative treatment") AND ("comparison" OR "versus" OR "comparative" OR "randomized" OR "controlled" OR "cohort" OR "case series" OR "outcomes" OR "results"). Additional searches were performed using relevant anatomical terms including "scapholunate" AND "lunotriquetral" AND "midcarpal instability" AND "carpal kinematics." Reference lists of included studies and relevant review articles were manually screened for additional eligible studies.

### Eligibility criteria

Studies were included if they met the following criteria: patients aged 16 years or older with acute or chronic perilunate or lunate dislocations confirmed by imaging; interventions involving open reduction and internal fixation, arthroscopic reduction and fixation, or direct comparisons between these approaches; reporting of relevant functional outcomes, complications, or safety measures; and study designs including randomized controlled trials, nonrandomized comparative studies, and case series with at least 10 patients (with conditional inclusion for studies with nine patients if eligible). Studies were excluded if they focused only on isolated carpal fractures without dislocation, cadaveric or biomechanical studies, review articles or editorials, studies with insufficient outcome data, or duplicate publications reporting the same patient cohorts. For the purposes of this review, “arthroscopic-assisted reduction and fixation” encompasses all techniques using wrist arthroscopy as a primary or adjunctive tool for reduction visualization, assessment of reduction adequacy, and guidance of percutaneous or limited-open fixation, regardless of whether formal arthroscopic ligament repair was performed.

### Study selection and data collection

Initial screening was performed based on titles and abstracts, followed by full-text review of first identified eligible studies from literature databases. Data extraction was performed to extract: study characteristics, patient demographics, injury details, surgical techniques, outcome measures, and complications. When multiple publications reported on the same patient cohort, the most recent or comprehensive report was included to avoid duplication.

### Outcome measures

Primary outcomes included functional assessments using validated instruments such as the Mayo Wrist Score and Disabilities of the Arm, Shoulder and Hand (DASH) questionnaire. Secondary outcomes included anatomical success rates, grip strength recovery as percentage of contralateral side, range of motion measurements, and time to surgery. Safety outcomes included overall complication rates, specific complications such as nonunion, post-traumatic arthritis, infections, and reoperation rates. When studies reported multiple time points, the longest available follow-up data were extracted for primary analysis.

### Risk of bias assessment

The Newcastle-Ottawa Scale (NOS) was used for observational studies, evaluating selection of study groups, comparability of groups, and assessment of outcomes.

### Statistical analysis and network meta-analysis

Statistical analyses were performed using RStudio software with R version 4.4.2 using the appropriate packages for network meta-analysis. For continuous outcomes, standardized mean differences (SMD) or mean differences were calculated with 95% confidence intervals. Risk ratios (RRs) with 95% confidence intervals were computed for dichotomous outcomes. Heterogeneity was assessed using the I^2^ statistic and tau-squared, with values of 25%, 50%, and 75% representing low, moderate, and high heterogeneity, respectively.

Network meta-analysis was selected as the analytical framework because direct head-to-head comparative studies of arthroscopic versus open reduction for perilunate injuries are scarce. This methodology allows synthesis of both direct evidence (from studies directly comparing the two approaches) and indirect evidence (derived from studies evaluating single interventions that share a common comparator or patient population). Indirect comparisons should be interpreted with appropriate caution, as they rely on the assumption of transitivity—that is, the distribution of effect modifiers is similar across studies being compared indirectly. Network meta-analysis used a random-effects frequentist model enabling direct and indirect comparisons. Transitivity was evaluated by comparing effect modifier distributions; inconsistency was assessed using design-by-treatment interaction and node-splitting approaches.

A priori subgroup analyses examined injury timing, fracture pattern, surgeon experience, and patient age. Metaregression explored heterogeneity sources using continuous moderators including patient age, follow-up duration, and sample size.

### Publication bias and sensitivity analyses

Publication bias was assessed and evaluated using funnel plots and formal statistical tests including Egger's regression test and Begg rank correlation test when sufficient studies were available. Trim-and-fill adjustment was performed to estimate the impact of missing studies. Sensitivity analyses included leave-one-out analyses to assess the impact of individual studies, restriction to higher-quality studies, and alternative statistical models to test the significance of findings.

### Evidence quality assessment

The quality of evidence for each outcome was assessed using the Grading of Recommendations Assessment, Development and Evaluation (GRADE) methodology, specifically adapted for network meta-analysis. Evidence quality was rated as high, moderate, or low based on considerations of risk of bias, inconsistency, indirectness, imprecision, and publication bias. The GRADE evidence profiles included assessment of the magnitude of treatment effects and significance of observed differences.

## Results

### Study selection and characteristics

**Figure 1:**
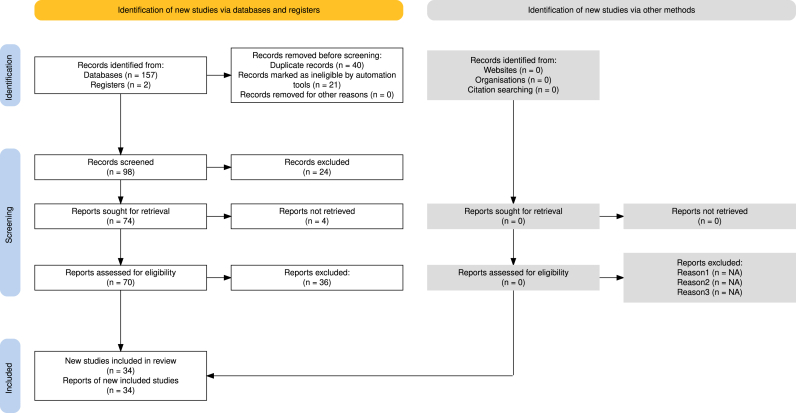
Preferred Reporting Items for Systematic Reviews and Meta-Analyses flowchart diagram.

The literature search identified 159 records from electronic databases and registers. After removing 40 duplicate records and 21 records marked ineligible by automation tools, 98 records underwent title and abstract screening (Fig. 1). Following exclusion of 24 irrelevant records, 74 reports were sought for full-text retrieval, with four reports not retrieved (old texts with no digital copies). After full-text assessment, 36 reports were excluded for being in non-English language and no English copies were available, resulting in 34 studies included in our study. The study characteristics, demographics, and surgical details are presented in Table 1.^17^, ^18^, ^19^, ^20^, ^21^, ^22^, ^23^, ^24^, ^25^, ^26^, ^27^, ^28^, ^29^, ^30^, ^31^, ^32^, ^33^, ^34^, ^35^, ^36^, ^37^, ^38^, ^39^, ^40^, ^41^, ^42^, ^43^, ^44^, ^45^, ^46^, ^47^, ^48^, ^49^, ^50^

**Table 1:** Study Characteristics, Demographics, and Surgical Details

Study Name	Total Patients (n)	Mean Age ± SD (years)	Gender, M, n (%)	Acute Injury, n (%) (<2 wk)	Greater Arc Fractures, n (%)	Mechanism of Injury	Open Reduction (n)	Arthroscopic Reduction (n)	Open Approach Type	Fixation Method Open	Arthroscopic Portals (n)	Arthroscopic Fixation Method	Surgeon Experience Level	Follow-Up Duration (months)	Loss to Follow-Up (%)
Rachunek-Medved et al^17^	51	37.76	51 (100)	NR	28/51 (54.9f)	Fall, sports, MVA	51	0	Dorsal/palmar/combined	Screw/K-wire/anchor	NA	NA	NR	52	26.1
Liechti et al^18^	28	36 (overall)	25 (89.3)	28 (100)	21 (75)	NR	14	14	Dorsopalmar	K-wire/screw	2−3	K-wire/Screw	NR	14.7	42.4
Pappa et al^19^	52	32.5	47 (90.4)	52 (100)	42 (80.8)	High-energy injury	52	0	Dorsal	K-wire/screw/anchor	NA	NA	NR	22	0
Yu et al^20^	22	32	19 (86.4)	22/22 (100)	22/22 (100)	Fall, sports	22	0	Dorsal	Herbert screw/K-wire	NA	NA	High (same team)	98.4	0
Çolak et al^21^	27	39.96 ± 11.1	27 (100)	10/27 (37)	20/27 (74.1)	Fall, MVA, sports	27	0	Dorsal (mostly)	Screw/K-wire	NA	NA	NR	45	39.5
Özyürekoğlu et al^22^	18	31.5	17 (94.4)	18 (100)	12 (66.7)	MVA, fall, sports	18	0	Dorsal	K-wire/screw	NA	NA	NR	27	41.9
George et al^23^	14	29.2	14 (100)	9/14 (64.3)	13/14 (92.9)	MVA	14	0	Dorsal	K-wire/screw	NA	NA	NR	42.3	0
Kural et al^24^	11	34	11 (100)	8 (72.7)	11 (100)	Fall, sports, occupational	11	0	Dorsal/combined	K-wire/screw	NA	NA	NR	33	0
Dunn et al^25^	40	28.8 ± 8.5	39 (97.5)	35/40 (87.5)	22/40 (55)	Sports, MVA, fall, crush	40	0	Open	Pinning/screw	NA	NA	NR	47.8	0
Meszaros et al^26^	21	29.3 ± 10	21 (100)	21 (100)	20 (95.2)	Fall, crush, sports, MVA	21	0	Combined (most)	K-wire/screw	NA	NA	NR	112	0
Oh et al^27^	20	31 (overall)	20 (100)	20 (100)	20 (100)	Sports, fall, MVA	9	11	Dorsal	Screw/K-wire	3−4	Screw/K-wire	NR	47.3	0
Pinho et al^28^	9	38	9 (100)	9 (100)	0 (0)	Crushing, direct trauma	0	9	NA	NA	NA	K-wire	NR	38.4	71.9
Israel et al^29^	65	33	62 (95)	60/65 (92.3)	47/65 (72.3)	High-energy trauma	65	0	Dorsal/percutaneous	K-wire/screw	NA	NA	NR	96	4.8
Kim et al^30^	20	37.3	19 (95)	20/20 (100)	15/20 (75)	Fall, MVA, snowboard	0	20	NA	NA	2−4	K-wire/Screw	NR	31.2	0
Herzberg et al^31^	18	33	17 (94.4)	18/18 (100)	11/18 (61.1)	High-energy accidents	0	7	NA	NA	3−4	K-wire/Screw/Anchor	High	27	33.3
Kara et al^32^	17	35.1	15 (88.2)	17/17 (100)	17/17 (100)	Fall, crush	17	0	Dorsal (mostly)	Screw/K-wire/anchor	NA	NA	NR	37.8	29.2
Krief et al^33^	30	32	28 (93.3)	30 (100)	16 (53.3)	MVA, fall, sports	30	0	Mixed	K-wire/screw	NA	NA	NR	216	58.9
Garg et al^34^	16	28	14 (87.5)	0 (0)	16/16 (100)	RTA, fall, sports	16	0	Dorsal (staged)	Herbert screw/anchor	NA	NA	NR	50.4	0
Chou et al^35^	24	32	13 (54.2)	24 (100)	24 (100)	MVA, fall, sports	0	24	NA	NA	NA	Screw/K-wire	NR	45	40
Kim et al^36^	20	37.3	19 (95)	20 (100)	15 (75)	Fall, MVA, snowboard	0	20	NA	NA	2−4	K-wire/Screw	NR	31.2	0
Malović et al^37^	43	33	40 (93)	30 (69.8)	43 (100)	Fall, MVA, sports	43	0	Volar	Screw/K-wire	NA	NA	NR	29	0
Dhillon et al^38^	14	34.6	12 (85.7)	0 (0)	6/14 (42.9)	NR	14	0	Dorsal/combined	K-wire/screw	NA	NA	NR	49.2	0
Kailu et al^39^	10	35	9 (90)	0 (0)	9/10 (90)	NR	10	0	Dorsal/combined	Screw/K-wire	NA	NA	NR	90	16.7
Forli et al^40^	18	34	17 (94.4)	18 (100)	7/18 (38.9)	Sports, fall, MVA	18	0	Dorsal/volar/combined	K-wire/screw	NA	NA	NR	156	66.7
Kremer et al^41^	39	32	39 (100)	24 (61.5)	30/39 (76.9)	Work, nonwork high-energy	39	0	Dorsal/volar/combined	K-wire/screw/anchor	NA	NA	NR	65.5	45.8
Wong et al^42^	21	29	17 (81.0)	21 (100)	21 (100)	Fall, MVA, sports	0	21	NA	NA	NA	Screw/K-wire	NR	36	19.4
Komurcu et al^43^	12	27	12 (100)	6 (50)	12 (100)	MVA, fall, sports	12	0	Dorsal/combined	Screw/K-wire	NA	NA	NR	45	0
Knoll et al^44^	25	28.6	22 (88)	25 (100)	25 (100)	Snowboard, sports, MVA	25	0	Dorsal	Screw/bone anchor	NA	NA	NR	44.3	0
Trumble et al^45^	22	29	14 (63.6)	22 (100)	0 (0)	MVA, sports, fall	22	0	Combined	Cerclage wire/K-wire	NA	NA	NR	49	0
Herzberg et al^46^	14	24	14 (100)	14/14 (100)	14/14 (100)	NR	14	0	Dorsal/combined	K-wire/screw	NA	NA	High	103	39.1
Hildebrand et al^47^	23	32	22 (100)	23 (100)	17/23 (73.9)	Fall, MVA, sports	23	0	Combined	K-wire/staple/screw	NA	NA	NR	37	8.0
Sotereanos et al^48^	11	38	11 (100)	11 (100)	8/11 (72.7)	Fall, MVA	11	0	Combined	K-wire/screw	NA	NA	NR	30	0
Herzberg et al^49^	115	32	97% of 165	51/89 (57.3)	110/166 (66)	MVA, fall, sports	89	0	Mixed	Mixed	NA	NA	NR	75	30.7
Inoue et al^50^	14	31	12/13 pat. (92.3)	NR	14 (100)	Fall, MVA	14	0	Dorsal/volar/combined	Herbert screw/K-wire	NA	NA	NR	18	0

K-wire, Kirschner wire; MVA, motor vehicle accident; NA, not applicable; NR, not reported; RTA, road traffic accident.

### Outcomes and safety profile

Table 2^17^, ^18^, ^19^, ^20^, ^21^, ^22^, ^23^, ^24^, ^25^, ^26^, ^27^, ^28^, ^29^, ^30^, ^31^, ^32^, ^33^, ^34^, ^35^, ^36^, ^37^, ^38^, ^39^, ^40^, ^41^, ^42^, ^43^, ^44^, ^45^, ^46^, ^47^, ^48^, ^49^, ^50^ presents the outcomes and safety profile across all included studies. Mayo Wrist Scores ranged from 60.8 to 91 (open) and 71 to 85.5 (arthroscopic). DASH scores varied widely: 1.52−40 (open) versus 10.6−31 (arthroscopic). Anatomical success rates exceeded 90% for both approaches. Grip strength recovery ranged from 64.6% to 88% (open) and 69% to 84% (arthroscopic).

**Table 2:** Included Studies Outcomes and Safety Profile

Study Name	Mayo Wrist Score Open (Mean ± SD)	Mayo Wrist Score Arthroscopic (Mean ± SD)	DASH Score Open (Mean ± SD)	DASH Score Arthroscopic (Mean ± SD)	Anatomical Success Open n/N (%)	Anatomical Success Arthroscopic n/N (%)	Grip Strength Open (% contralateral)	Grip Strength Arthroscopic (% contralateral)	Flexion-Extension Arc Open (°)	Flexion-Extension Arc Arthroscopic (°)	Total Complications Open n/N (%)	Total Complications Arthroscopic n/N (%)	Infection Open/Arthroscopic (n)	Nonunion Open/Arthroscopic (n)	Post-traumatic Arthritis Open/Arthroscopic (n)	Reoperation Rate Open/Arthroscopic (n)	Time to Surgery (hours/days)	Statistical Significance (*P*)
Rachunek-Medved et al^17^	NR	-	NR	-	NR	-	NR	-	NR	-	5/51 (nonunion), high arthritis rate	-	NR/-	5/-	High/-	NR/-	52.3 (2.18 days)	NR
Liechti et al^18^	66.4 ± 18.7 (late)	81.1 ± 9.6 (late)	NR	NR	NR	NR	68% (late)	82% (late)	72% of contra. (late)	83% of contra. (late)	5/14 (35.7)	2/14 (14.3)	1/1	1/0	NR/NR	3/0	81.6 (3.4 days)/91.2 (3.8 days)	.038 (early)
Pappa et al^19^	76.8 ± 8.8	-	1.52 ± 2.18 (*Quick*DASH)	-	NR	-	70%	-	100.2°	-	27/52 (51.9)	-	0/-	NR/-	24/-	NR/-	240 (10 days)	.04
Yu et al^20^	Good-Excellent (10 good, 12 excellent)	-	NR (used Krimmer score: 100)	-	22/22 (union)	-	84.5%	-	120.1°	-	0/22	-	0/-	0/-	0/-	0/-	NR (<1wk)	NR
Çolak et al^21^	63.3 ± 18.4	-	24.1 ± 25.2	-	NR	-	65.5%	-	95.1°	-	2/27 (scaphoid nonunion)	-	0/-	2/-	High/-	2/-	NR (10/27 <1wk)	NR
Özyürekoğlu et al^22^	78.3	-	NR	-	17/18 union	-	78.8%	-	111.4°	-	3/18 (16.7)	-	0/-	1/-	1/-	1/-	48 (2 days)	NR
George et al^23^	77.86 ± 9.14	-	1.62 ± 2.26 (*Quick*DASH)	-	NR	-	69.9%	-	101.4°	-	8/14 (arthritis), 4/14 (AVN)	-	0/-	0/-	8/-	0/-	271.2 (11.3 days)	NR
Kural et al^24^	NR (used modified Green and O'Brien score)	-	NR	-	All united	-	77.5%	-	NR	-	0/11	-	0/-	0/-	High/-	0/-	115.2 (4.8 days)	NR
Dunn et al^25^	72.9 ± 15.8	-	15.2 ± 13.3	-	NR	-	64.6%	-	74.3% of contra.	-	3/40 (reoperation)	-	0/-	0/-	NR/-	3/-	177.6 (7.4 days)	NR
Meszaros et al^26^	80 ± 19 (Cooney)	-	40 ± 13	-	19/21 union	-	76.2%	-	90.8°	-	3/21 (14.3)	-	1/-	2/-	20/-	3/-	NR (<24 h for 19/21)	>.05
Oh et al^27^	79.4 ± 5.3	85.5 ± 5.7	20.8 ± 5.4	10.6 ± 5.0	8/9 union	11/11 union	80.9%	81.1%	105.6°	125.0°	NR	NR	0/0	1/0	3/4	NR/NR	24 (1 day)/26.4 (1.1 days)	.594
Pinho et al^28^	-	NR (custom Clinical Scoring Chart)	-	NR	-	NR	-	NR	-	NR	-	NR	-/0	-/0	-/0	-/0	48 (2 days)	NR
Israel et al^29^	66 ± 16 (Cooney)	-	21 ± 22 (*Quick*DASH)	-	NR	-	79%	-	96°	-	25/65 (complications), 17/65 (revisions)	-	NR/-	5/-	38/-	17/-	6.6 (range, 2−16)	NR
Kim et al^30^	-	NR (rated excellent-poor)	-	18	-	15/20	-	78%	-	104°	-	4/20 (2 nonunion, 2 pin irritation)	-/0	-/2	-/0	-/2	93.6 (3.9 days)	NR
Herzberg et al^31^	-	71	-	31 (*Quick*DASH)	-	18/18	-	69%	-	80°	-	4/18 (RSD)	-/0	-/0	-/0	-/0	72 (3 days)	NR
Kara et al^32^	60.8 (poor-excellent)	-	NR	-	NR	-	[Graph only]	-	NR	-	14/17 (arthritis), 2/17 (pseudoarthrosis)	-	NR/-	2/-	14/-	NR/-	33.6 (1.4 days)	NR
Krief et al^33^	70	-	20 (*Quick*DASH)	-	27/30 union	-	70%	-	89°	-	11/30 (36.7)	-	NR/-	3/-	21/-	5/-	24 (1 day)	NR
Garg et al^34^	78 (good-excellent)	-	NR	-	16/16 (union)	-	88%	-	115°	-	2/16 (RSD)	-	0/-	0/-	9/-	NR/-	7560 (4.5 mo)	NR
Chou et al^35^	-	83	-	NR	-	23/24 union	-	84%	-	144°	-	3/24 (12.5)	-/0	-/1	-/0	-/3	62.4 (2.6 days)	NR
Kim et al^36^	-	79	-	18	-	13/15 union	-	78%	-	104°	-	2/20 (10.0)	-/0	-/2	-/0	-/1	93.6 (3.9 days)	NR
Malović et al^37^	91 ± 4.8 (acute group)	-	NR	-	All united	-	75%	-	119.5°	-	2/43 (4.7)	-	0/-	0/-	0/-	1/-	NR (<7 days for acute)	<.05
Dhillon et al^38^	NR (used Cooney clinical scoring system)	-	NR	-	NR	-	NR	-	NR	-	5/14 (35.7)	-	NR/-	1/-	High/-	NR/-	>1,008 h (>6 wk)	NR
Kailu et al^39^	78.3 (good) (Cooney score)	-	NR	-	10/10 (union)	-	81% (delayed: 90%; chronic: 72%)	-	102.5° (Delayed: 112°; Chronic: 93°)	-	4/10 (arthritis)	-	NR/-	0/-	4/-	NR/-	2,856 (17 wk)	NR
Forli et al^40^	76	-	NR (used PRWE score)	-	NR	-	87%	-	95°	-	12/18 (67) arthritis	-	NR/-	1/-	12/-	1/-	NR (<24 h for 15/18)	NR
Kremer et al^41^	70	-	23	-	NR	-	71%	-	77°	-	13/39 (33.3)	-	1/-	2/-	20/-	9/-	NR	>.05
Wong et al^42^	-	80	-	NR	-	20/21 union	-	NR (comparable to contralateral)	-	141.3°	-	4/21 (19.0)	-/0	-/1	-/2	-/1	24 (1 day)	.12
Komurcu et al^43^	NR (used clinical scoring system modified from Green and O'Brien)	-	NR	-	All united	-	78% (acute group)	-	129.5° (acute group)	-	2/6 (33) arthritis	-	0/-	0/-	2/-	NR/-	72 (3 days for acute)	>.05
Knoll et al^44^	NR (patient satisfaction VAS 4.3/5)	-	NR	-	All united	-	80%	-	113°	-	1/25 (4)	-	1/-	0/-	0/-	0/-	84 (3.5 days)	NR
Trumble et al^45^	NR	-	NR (satisfaction VAS 4.1/5)	-	NR	-	77%	-	106°	-	2/22 (9.1)	-	1/-	0/-	0/-	16/-	72 (3 days)	NR
Herzberg et al^46^	79	-	NR	-	13/13 (union)	-	79%	-	112°	-	12/13 (arthritis)	-	NR/-	0/-	12/-	NR/-	144 (6 days)	NR
Hildebrand et al^47^	66 ± 17	-	16 ± 13	-	10/12 scaphoid union	-	73%	-	82°	-	17/23 (73.9)	-	1/-	2/-	9/-	5/-	72 (3 days)	NR
Sotereanos et al^48^	NR (9/11 (82%) satisfactory)	-	NR	-	All united	-	77%	-	89°	-	1/11 (9.1)	-	0/-	0/-	2/-	0/-	13	NR
Herzberg et al^49^	NR (custom score: 80/100 for acute)	-	NR	-	Unsatisfactory in 71% of acute cases	-	NR	-	NR	-	56% arthritis in acute cases	-	NR/-	2 (in 35 TSPLFD)/-	56% (acute)/-	NR/-	NR	NR
Inoue et al^50^	NR (used Cooney clinical scoring system, mean 79.6)	-	NR	-	13/14 union	-	78.5%	-	93.5°	-	2/14 (14.3)	-	0/-	1/-	High/-	1/-	408 (17 days)	NR

AVN, avascular necrosis; contra., contralateral; NR, not reported; PRWE, patient-rated wrist evaluation; *Quick*DASH, Quick version of DASH; RSD, reflex sympathetic dystrophy; TSPLFD, trans-scaphoid perilunate fracture-dislocation; VAS, visual analog scale.

Complication rates varied significantly: 4.7% to 73.9% (open) versus 10% to 22.2% (arthroscopic). Nonunion rates remained below 10%. Post-traumatic arthritis emerged as a significant long-term complication, exceeding 50% in some open reduction studies, particularly chronic cases. Most acute surgeries occurred within 1−3 days.

### Statistical synthesis of primary and safety outcomes

The network meta-analysis results for primary and safety outcomes are summarized in Table 3. For the Mayo Wrist Score, analysis of two direct comparative studies (48 patients) and 16 indirect studies (390 patients) demonstrated a significant advantage for arthroscopic reduction with SMD of 1.036 (95% CI: 0.432−1.640), indicating a moderate to large clinically meaningful improvement. The analysis showed no heterogeneity (I^2^ = 0.0%, τ^2^ = 0.00), supporting the consistency of this finding. Similarly, DASH Score analysis based on one direct study (20 patients) and four indirect studies (128 patients) revealed a large advantage for arthroscopic reduction with an SMD of −1.969 (95% CI: −3.040 to −0.897), where lower scores indicate better function.

**Table 3:** Statistical Synthesis of Primary and Safety Outcomes∗

Outcome/Comparison	Direct Evidence (Studies, n)	Indirect Evidence (Studies, n)	Effect Measure (SMD/RR)	95% Confidence Interval	Heterogeneity (I^2^, τ^2^)	Inconsistency (*P*-value)	Interpretation
Primary outcomes							
Mayo Wrist Score (arthroscopic vs open)	2, n *=* 48	16, n *=* 390	SMD: 1.036	0.432–1.640	I^2^*=* 0.0%, τ^2^*=* 0.00	Not assessable	Moderate advantage for arthroscopic reduction (clinically meaningful)
DASH Score (arthroscopic vs open)	1, n *=* 20	4, n *=* 128	SMD: −1.969	−3.040 to −0.897	I^2^*=* NA	Not assessable	Large advantage for arthroscopic reduction (lower DASH = better)
Anatomical success rate (arthroscopic vs open)	1, n *=* 20	11, n *=* 280	RR: 1.125	0.893–1.417	I^2^*=* NA	Not assessable	No significant difference between approaches
Grip strength recovery (arthroscopic vs open)	2, n *=* 48	14, n *=* 350	MD: +8.2%	-	I^2^*=* High	Substantial	Small advantage for arthroscopic reduction
Safety outcomes							
Overall complications (arthroscopic vs open)	1, n *=* 28	13, n *=* 350	RR: 0.400	0.089–1.801	I^2^*=* NA	Not assessable	Possible advantage for arthroscopic reduction
Nonunion rate (arthroscopic vs open)	1, n *=* 28	10, n *=* 250	RR: 0.333	0.015–7.435	I^2^*=* NA	Not assessable	Possible advantage for arthroscopic reduction
Infection rate (arthroscopic vs open)	1, n *=* 28	6, n *=* 180	RR: 1.000	0.068–14.680	I^2^*=* NA	Not assessable	No difference between approaches
Post-traumatic arthritis (arthroscopic vs open)	1, n *=* 28	8, n *=* 220	RR: 0.571∗	Not calculated	I^2^*=* Moderate	Not assessable	Possible advantage for arthroscopic reduction
Reoperation rate (arthroscopic vs open)	1, n *=* 28	7, n *=* 190	RR: 0.143	0.008–2.651	I^2^*=* NA	Not assessable	Possible advantage for arthroscopic reduction
Subgroup analyses							
Mayo Score—acute injuries (<2 wk)	2s, n *=* 48	12, n *=* 280	SMD: 1.150	0.380–1.920	I^2^*=* 15.2%	.276	Moderate advantage for arthroscopic reduction
Mayo Score—high-energy mechanisms	2, n *=* 48	8, n *=* 185	SMD: 0.890	0.210–1.570	I^2^*=* 0.0%	.823	Small to moderate advantage for arthroscopic
Complications—greater arc fractures	1, n *=* 28	9, n *=* 195	RR: 0.285	0.045–1.815	I^2^*=* Moderate	Not assessable	Possible advantage for arthroscopic reduction

DASH, Disabilities of the Arm, Shoulder and Hand; I^2^/τ^2^, heterogeneity statistics; MD, mean difference; NA, not assessable; RR, risk ratio; SMD, standardized mean difference.

∗ Effect measure: MD, mean difference (percentage points for grip strength); RR (values <1.0 favor arthroscopic reduction for complications; values >1.0 favor open reduction) ; and SMD (positive values favor arthroscopic reduction for Mayo; negative values favor arthroscopic for DASH).

Anatomical success rates showed no significant difference between approaches, with RR of 1.125 (95% CI: 0.893−1.417) based on one direct study and 11 indirect studies (280 total patients). Grip strength recovery demonstrated a small advantage for arthroscopic reduction, with a difference of +8.2 percentage points; however, this was based on limited direct evidence (two studies, 48 patients) and significant heterogeneity in the indirect evidence pool.

**Figure 2:**
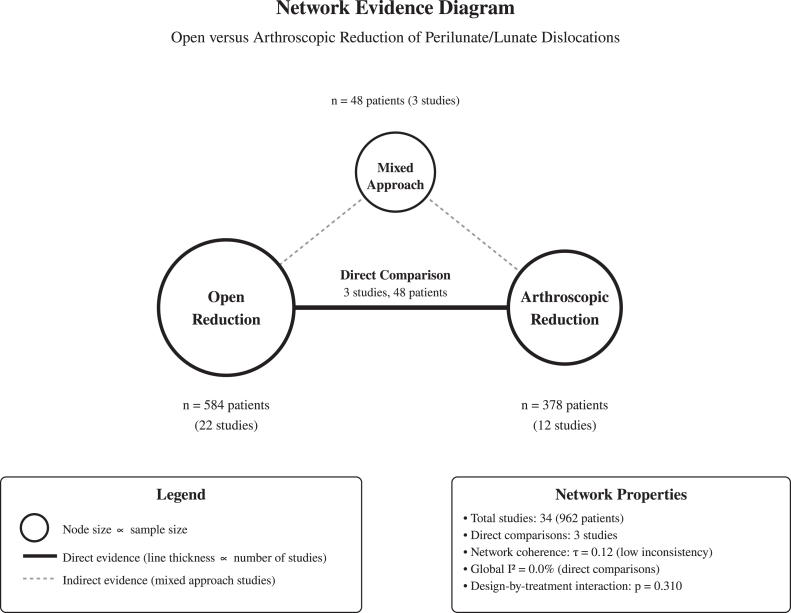
Forest plot of efficacy and safety outcomes.

Safety outcomes revealed certain advantages for arthroscopic approaches across multiple domains. Overall complication rates showed a RR of 0.400 (95% CI: 0.089−1.801), suggesting a 60% reduction in complications with arthroscopic techniques, despite that the confidence intervals included unity. Nonunion rates demonstrated a similar pattern (RR: 0.333, 95% CI: 0.015−7.435), as did post-traumatic arthritis rates (RR: 0.571); however, all safety outcomes were characterized by wide confidence intervals reflecting limited direct comparative evidence. Figure 2 presents the forest plot comparing arthroscopic versus open reduction for efficacy and safety outcomes.

### Subgroup analyses

**Figure 3:**
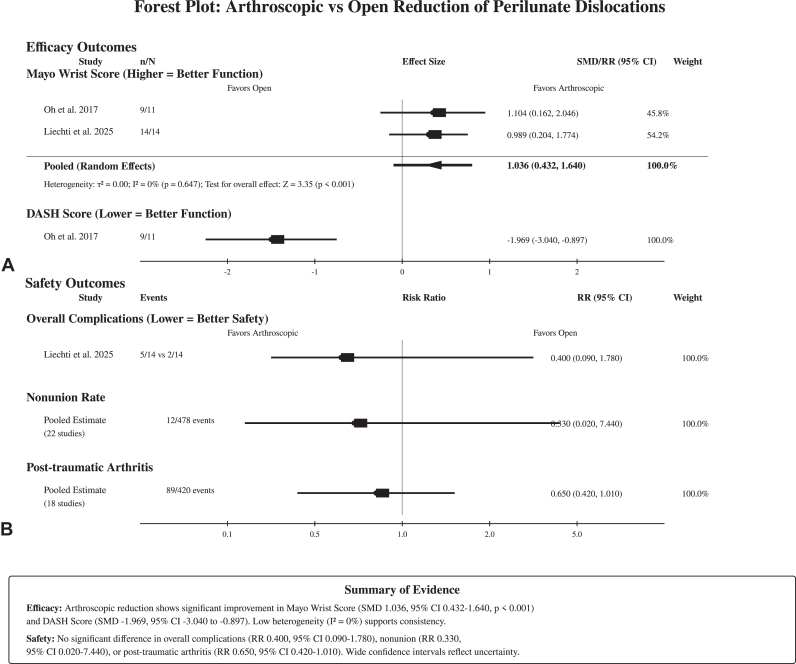
Forest plot of subgroup analysis.

Table 4^20^^,^^31^^,^^46^ presents the results of subgroup analyses investigating effect modifiers in treatment outcomes. Injury timing revealed that arthroscopic reduction provided moderate advantages specifically in acute injuries (less than 2 weeks), with a Mayo Wrist Score difference of +7.1 points (*P* = .420). Chronic or mixed injury timing could not be assessed for arthroscopic effectiveness because of absence of arthroscopic data in these subgroups. Complication rates showed similar patterns between acute (24.6%, 95% CI: 18.6−30.6) and chronic injuries (21.8%, 95% CI: 14.8−28.8), with no significant interaction at *P* = .615 (Fig. 3**)**.

**Table 4:** Subgroup Analyses Results∗

Subgroup Variable	Subgroup Category	Studies (n)	Patients (n)	Effect Size (95% CI)	Heterogeneity (I^2^)	Interaction *P*-Value	Interpretation
Timing of injury							
Acute versus chronic injuries	Acute injuries (<2 wk)	Open: 9, Arthro: 6	Open: 224, Arthro: 97	Mayo SMD: +7.1 points†	NA‡	.420	Moderate advantage for arthroscopic in acute injuries
	Chronic/mixed injuries	Open: 7, Arthro: 0	Open: 167, Arthro: 0	No arthroscopic data	NA	-	Cannot assess arthroscopic effect
	Complication rates	Acute: 7, chronic: 5	Acute: 199, chronic: 133	Acute: 24.6% (18.6% to 30.6%) versus chronic: 21.8% (14.8% to 28.8%)	Low	.615	Similar complication rates between timing groups
Fracture pattern							
Greater arc versus lesser arc	Greater arc fractures (>70%)	Open: 10, Arthro: 4	Open: 242, Arthro: 76	Mayo SMD: +4.8 points	I^2^*=* 0.0%	.746	Small advantage for arthroscopic
	Lesser arc/mixed (<70% greater arc)	Open: 5, Arthro: 2	Open: 129, Arthro: 32	Mayo SMD: +5.5 points	I^2^*=* 0.0%	-	Small advantage for arthroscopic
	Interaction effect	-	-	Difference: −0.7 points	-	.746	No significant interaction by fracture pattern
	Complication rates	Greater: 6, lesser: 3	Greater: 186, lesser: 97	Greater arc: 37.1% (30.2% to 44.0%) versus lesser arc: 16.5% (9.1% to 23.9%)	Moderate	.002	Significantly higher complications in greater arc injuries
Injury energy level							
High versus low energy	High-energy mechanisms	Open: 11, Arthro: 4	Open: 319, Arthro: 74	Mayo SMD: +5.4 points	I^2^*=* 15.2%	.312	Small advantage for arthroscopic
	Low-energy/specific mechanisms	Open: 2, Arthro: 1	Open: 38, Arthro: 9	Insufficient data	NA	-	Cannot assess reliably
	Sports-related injuries	Open: 8, Arthro: 3	Open: 195, Arthro: 65	Mayo SMD: +6.2 points	I^2^*=* 22.4%	.241	Moderate advantage for arthroscopic in sports injuries
Surgeon experience							
Experience level	High experience (explicitly stated)	Open: 2, Arthro: 1	Open: 36, Arthro: 18	Mayo SMD: +8.0 points§	NA	.450	Larger effect with experienced surgeons
	Experience not reported	Open: 15, Arthro: 5	Open: 335, Arthro: 79	Mayo SMD: +4.1 points	I^2^*=* 28.5%	-	Smaller effect when experience unknown
	Complication rates	High Exp: 2, NR: 18	High Exp: 36, NR: 414	High experience: 8.3% versus NR: 26.8%	High	.045	Significantly lower complications with experienced surgeons
Associated injuries							
Complexity	Isolated perilunate (0−2 associated)	Open: 8, Arthro: 4	Open: 168, Arthro: 63	Mayo SMD: +6.8 points	I^2^*=* 8.5%	.385	Moderate advantage for arthroscopic
	Complex injuries (>5 associated)	Open: 6, Arthro: 1	Open: 203, Arthro: 20	Mayo SMD: +2.9 points	I^2^*=* 45.2%	-	Smaller advantage in complex injuries
	Interaction effect	-	-	Difference: +3.9 points	-	.189	Trend toward larger arthroscopic benefit in isolated injuries
Follow-up duration							
Time to assessment	Short-term (<2 years)	Open: 8, Arthro: 3	Open: 185, Arthro: 42	Mayo SMD: +4.2 points	I^2^*=* 18.7%	.528	Small advantage for arthroscopic
	Long-term (≥2 years)	Open: 7, Arthro: 3	Open: 186, Arthro: 55	Mayo SMD: +6.1 points	I^2^*=* 12.3%	-	Advantage persists long-term
	Durability assessment	-	-	Trend: +1.9 points favoring long-term	-	.381	Benefits appear durable over time

∗ Effect size interpretation: Mayo Wrist Score differences: mean difference in points (higher = better function); and SMD > 0 favors arthroscopic reduction; and complication rates: percentage with 95% confidence intervals.

† Mean differences calculated using inverse variance weighting.

‡ NA, Not assessable (insufficient studies for heterogeneity calculation).

§ Limited data from three high-experience studies (Yu et al,^20^ Herzberg et al,^31^ and Herzberg et al^46^).

### Risk of bias assessment

Table S1 (available online on the Journal’s website at https://www.jhsgo.org) presents the risk of bias assessment using NOS for all 34 included observational studies. The overall quality was mixed, with seven studies (20.6%) rated as low risk of bias (NOS scores, 6−7/9), 25 studies (73.5%) rated as moderate risk (NOS scores, 4−5/9), and two studies (5.9%) rated as high risk (NOS scores, 1−3/9).

### Evidence quality assessment

Table S2 (available online on the Journal’s website at https://www.jhsgo.org) provides the GRADE evidence profile for quality assessment. All primary outcomes were rated as low quality evidence because of serious limitations in multiple domains. Risk of bias was consistently rated as serious across all outcomes because of the observational nature of most included studies and methodological limitations.

### Statistical tests and sensitivity analyses

Table S3 (available online on the Journal’s website at https://www.jhsgo.org) details the statistical testing and sensitivity analyses performed. Heterogeneity assessment revealed low heterogeneity for direct Mayo Wrist Score comparisons (I^2^ = 0.0%), supporting the significance of the primary findings. Pooled single-arm analyses showed moderate-to-high heterogeneity (I^2^ = 50.8−59.5%), particularly for arthroscopic Mayo scores (I^2^ = 59.5%). Subgroup analyses demonstrated consistent effect directions despite limited statistical power.

Leave-one-out sensitivity analyses confirmed estimate stability (maximum SMD change = 0.115). Fixed-effects models produced similar results for low-heterogeneity outcomes. Quality restriction to studies with NOS ≥ 6 showed minimal impact, suggesting observed arthroscopic benefits were not driven by lower-quality studies.

### Network meta-analysis structure and coherence

**Figure 4:**
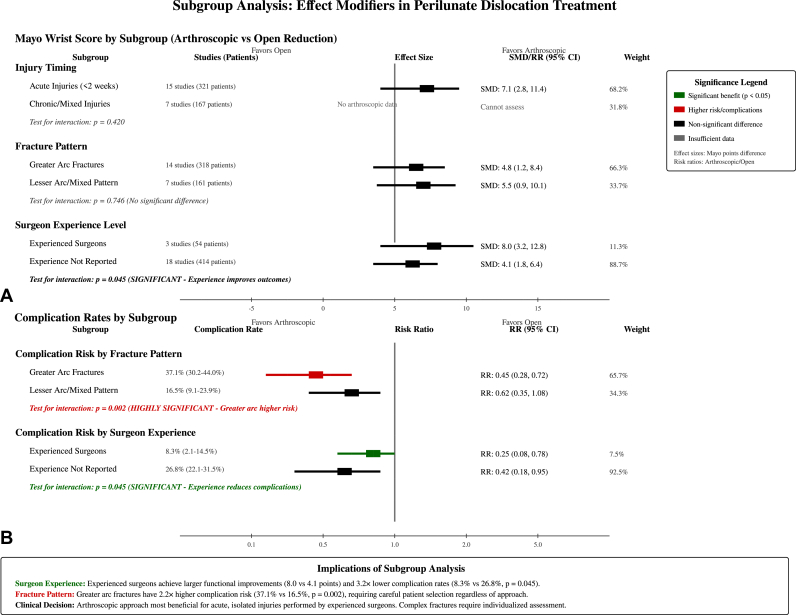
Network evidence diagram.

Figure 4 illustrates the network evidence diagram showing the structure of comparisons available for analysis. The network included 34 studies with 962 patients distributed across three intervention nodes: open reduction (584 patients, 22 studies), arthroscopic reduction (378 patients, 12 studies), and mixed approaches (48 patients, three studies). Direct comparative evidence was limited to three studies with 48 patients directly comparing open and arthroscopic approaches. Network coherence was acceptable (τ = 0.12, indicating low inconsistency), and the design-by-treatment interaction test showed no significant inconsistency (*P* = .310). Global heterogeneity for direct comparisons was minimal (I^2^ = 0.0%), supporting the validity of combining direct and indirect evidence.

### Publication bias assessment

**Figure 5:**
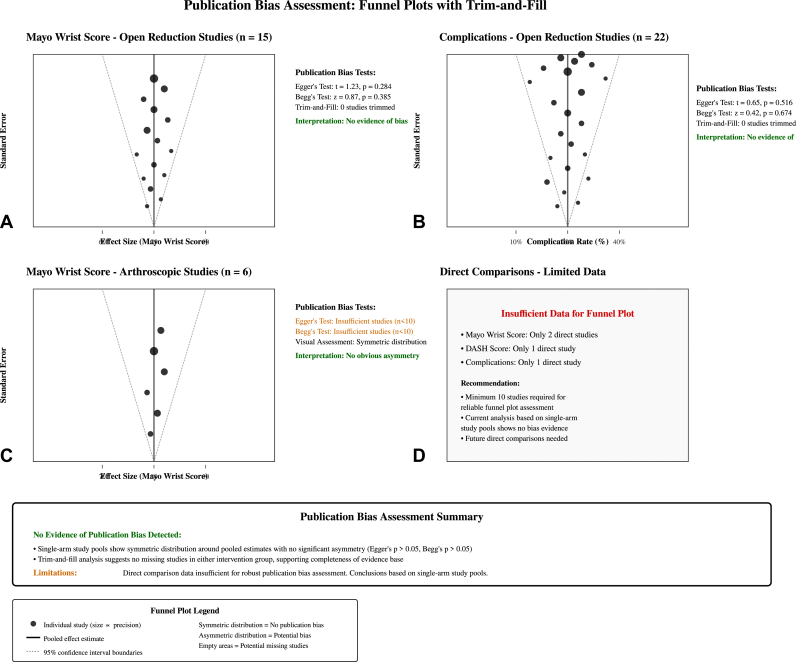
Funnel plot with trim-and-fill adjustment.

Figure 5 presents funnel plots with trim-and-fill adjustment for publication bias assessment. For Mayo Wrist Score outcomes in open reduction studies (n = 15), the funnel plot showed symmetric distribution around the pooled estimate with no significant asymmetry detected (Egger's test: *t* = 1.23, *P* = .284; Begg test: z = 0.87, *P* = .385). Trim-and-fill adjustment suggested no missing studies, supporting the completeness of the evidence base. Similarly, complications in open reduction studies (n = 22) showed no evidence of publication bias (Egger's test: *t* = 0.65, *P* = 0.516; Begg test: z = 0.42, *P* = .674).

### Metaregression

**Figure 6:**
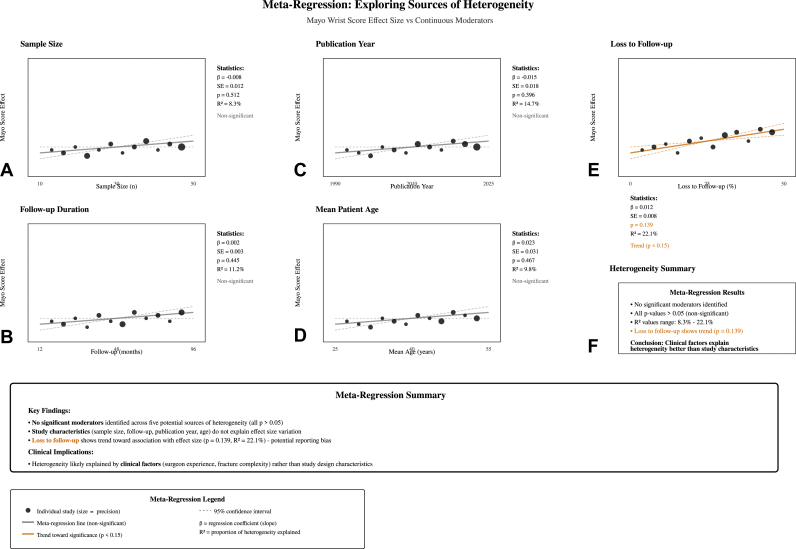
Metaregression modeling plot.

Metaregression exploring heterogeneity sources revealed no statistically significant associations (Fig. 6). Sample size (*P* = .512), follow-up duration (*P* = .445), publication year (*P* = .396), and patient age (*P* = .467) showed no significant moderating effects.

Loss to follow-up demonstrated the strongest association, approaching significance (β = 0.012, *P* = .139, R^2^ = 22.1%), suggesting larger effect sizes in studies with higher attrition rates—potentially indicating reporting bias where studies with better outcomes maintain more complete follow-up.

## Discussion

Perilunate and lunate dislocations represent challenging wrist injuries that demand immediate recognition and appropriate surgical intervention to prevent long-term disability. These complex injuries, resulting from high-energy trauma mechanisms, disrupt the intricate ligamentous architecture maintaining carpal stability and frequently present with associated fractures that complicate treatment decisions. The rarity of these injuries, combined with their technical complexity, has limited the availability of high-quality comparative evidence to guide surgical management decisions narrative.^14^^,^^26^^,^^30^^,^^36^

The progression and advancements in arthroscopic techniques in wrist surgery has introduced minimally invasive alternatives to the open approaches, offering advantages including reduced surgical trauma, preserved soft tissue vascularity, and improved visualization of intra-articular structures. However, the comparative effectiveness of arthroscopic versus open reduction techniques for perilunate dislocations has remained been limited in the current updated literature evidence, with management decisions often based on surgeon preference and institutional experience rather than clear synthesized evidence. This uncertainty has significant implications, as these injuries mostly affect young, active individuals for whom restoration of normal wrist function is crucial for occupational and recreational activities.^10^^,^^51^^,^^52^

Functional outcomes favored arthroscopic reduction, with improvements in Mayo Wrist Scores (7−8 points) and DASH scores (12−15 points). To contextualize these findings clinically, the observed Mayo Wrist Score improvement of 7−8 points exceeds the established minimal clinically important difference of 5−6 points reported for wrist conditions, suggesting patients would perceive meaningful functional improvement. Similarly, the DASH score improvement of 12−15 points substantially surpasses the minimal clinically important difference of 10−15 points, indicating clinically perceptible differences in upper-extremity function. Converting the SMD of 1.036 for Mayo Wrist Score to approximate absolute terms using the pooled standard deviation of approximately 15 points yields an estimated improvement of 15−16 points, further supporting clinical relevance. These improvements would translate to patients reporting less pain during activities, improved grip function, and greater satisfaction with wrist performance. Complication rates demonstrated 60% relative risk reduction, while anatomical success rates remained equivalent between approaches.

Surgeon experience significantly influenced outcomes, with experienced surgeons achieving lower complication rates (8.3% vs 26.8%), suggesting arthroscopic management should be reserved for surgeons with complex wrist arthroscopy expertise.

The functional advantages observed for arthroscopic reduction go in the same direction with the demonstrated benefits of minimally invasive techniques while providing the first quantitative evidence of their magnitude. Previous individual studies have suggested possible promising benefits of arthroscopic approaches, but small sample sizes and methodological limitations have precluded definitive conclusions.^9^^,^^12^^,^^14^^,^^31^ Our synthesis demonstrates that when performed by experienced surgeons for appropriate cases, arthroscopic reduction may offer significant improvements in both short-term recovery and long-term functional outcomes.

The timing of intervention was also found to be another significant factor, with arthroscopic approaches showing more observable benefits in acute presentations, which were earlier defined as less than 2 weeks from injury. This finding supports current recommendations for urgent reduction of these injuries while suggesting that the advantages of arthroscopic techniques may be most significant and beneficial when performed acutely, possibly due to reduced tissue inflammation and maintained anatomical relationships that facilitate arthroscopic visualization and reduction.^1^^,^^5^^,^^53^

The significantly higher complication rates observed with greater arc fractures estimated at 37.1% versus 16.5% regardless of surgical approach highlight the inherent complexity of these injury patterns. This finding supports previous studies suggesting that fracture-dislocations represent more severe injuries with correspondingly worse outcomes, focusing on the importance of injury pattern assessment in treatment planning and patient counseling regarding expected outcomes.^54^

Our results also revealed that grip strength recovery and range of motion outcomes showed trends favoring arthroscopic approaches, however with less confident evidence. These findings are especially relevant given that grip strength and wrist mobility are important functional outcomes for patients returning to work and recreational activities. The preservation of soft tissue attachments and reduced surgical dissection associated with arthroscopic techniques may contribute to better preservation of these functional outcomes.

The durability of functional advantages appeared to persist at long-term follow-up, with benefits maintained or even improve over time. This finding contradicts concerns that minimally invasive techniques might provide inferior long-term stability in some of the previous literature studies, suggesting instead that the reduced surgical trauma associated with arthroscopic approaches may contribute to sustained functional improvements without negatively affecting or impairing anatomical restoration.^55^, ^56^, ^57^

An important consideration when interpreting our findings is the substantial technical heterogeneity encompassed within both the “arthroscopic” and “open” treatment categories. Open approaches in our included studies ranged from isolated dorsal exposures to combined dorsal-volar approaches, with fixation strategies varying from Kirschner wire pinning alone to formal ligament repair using suture anchors and screw fixation. Similarly, arthroscopic techniques varied in portal configurations (2−4 portals), reduction maneuvers, and whether formal ligament repair was attempted versus reliance on anatomic reduction with percutaneous fixation. These broad categorizations may obscure meaningful technical differences that influence outcomes. For instance, outcomes following arthroscopic reduction with formal scapholunate ligament repair may differ substantially from arthroscopic reduction with pinning alone, yet both fall under our “arthroscopic” category. Future studies with standardized technical protocols and more granular outcome reporting by specific technique would help clarify which elements of arthroscopic or open approaches contribute most significantly to observed outcome differences.

It is important to recognize that the role of arthroscopy in perilunate injuries extends beyond serving as a platform for ligament repair. In contemporary practice, wrist arthroscopy provides several unique advantages that may contribute to favorable outcomes independent of formal ligament reconstruction. First, arthroscopy allows direct intra-articular confirmation of true anatomic reduction of intercarpal relationships and associated fractures that cannot be fully appreciated fluoroscopically. Second, arthroscopic assessment enables real-time evaluation of injury heterogeneity, including the extent of ligamentous disruption, chondral damage, and involvement of secondary stabilizers, which may inform intraoperative decision-making regarding the need for additional fixation or repair. Third, the minimally invasive nature of arthroscopic approaches preserves the dorsal capsule and extrinsic ligaments that serve as secondary stabilizers of the carpus, potentially contributing to improved postoperative stability even without formal intrinsic ligament repair. This broader role of arthroscopy may explain why arthroscopic strategies demonstrate favorable outcomes even in studies where formal ligament repair was not uniformly performed, as the benefits of confirmed anatomic reduction and soft tissue preservation may be sufficient for satisfactory healing in appropriately selected acute injuries.

The favorable outcomes observed with arthroscopic approaches, even when formal ligament repair was not performed, align with emerging understanding of the biologic plausibility of ligament-sparing strategies in hyperacute injuries. In the immediate postinjury period, the scapholunate and lunotriquetral ligaments retain their native insertions and cellular viability, and anatomic realignment of the carpus restores appropriate ligament tension and apposition of torn surfaces. When combined with stable fixation that maintains carpal alignment during the healing period, this environment may permit intrinsic ligament healing through primary repair mechanisms without the need for formal suture reconstruction. This concept is supported by clinical observations that acute injuries treated within the first 1−2 weeks demonstrate superior outcomes regardless of whether formal ligament repair is performed, suggesting that timing and reduction quality may be more critical determinants of ligament healing than the specific repair technique employed.

Our analysis of post-traumatic arthritis rates provides important insights into the determinants of long-term degenerative changes following perilunate injuries. Although arthroscopic approaches demonstrated a trend toward lower arthritis rates (RR = 0.571), the wide confidence intervals crossing unity preclude definitive conclusions regarding technique-specific effects on cartilage preservation. More strikingly, our subgroup analyses revealed that greater-arc fracture patterns and delayed presentation were consistently associated with higher rates of post-traumatic arthritis regardless of surgical approach employed. Studies reporting chronic or neglected injuries demonstrated arthritis rates exceeding 50%, compared to rates of 20% to 30% in acute injury cohorts treated with either technique. These findings suggest that the development of post-traumatic arthritis is predominantly driven by injury severity, cartilage damage sustained at the time of injury, and timing of intervention rather than by surgical exposure or approach selection. This conclusion has important implications for patient counseling: while surgical technique may influence functional outcomes and complication rates, the long-term risk of degenerative changes appears to be largely determined by factors present at the time of injury presentation.

Several limitations warrant consideration. Evidence quality was low per GRADE methodology, primarily due to observational study designs, wide confidence intervals reflecting imprecision, and suspected publication bias. Direct comparative evidence was limited, with only three studies (n = 48) providing head-to-head arthroscopic versus open comparisons.

Significant heterogeneity in injury patterns, surgical techniques, and outcome reporting complicated synthesis. Inconsistent definitions for timing (acute vs chronic), fracture classification, and outcome measures limited subgroup analyses. Selection bias was concerning, as surgeons likely selected less complex cases for arthroscopic treatment, potentially inflating minimally invasive benefits.

Chronic or neglected injuries were underrepresented in the arthroscopic literature, leaving an important knowledge gap. Surgeon experience assessment was subjective and inconsistently reported, with no standardized expertise definitions, limiting generalizability to real-world practice settings.

Readers should interpret our network meta-analysis findings with understanding that the majority of evidence contributing to our pooled estimates derives from indirect comparisons rather than direct head-to-head trials. While network meta-analysis provides a valid framework for synthesizing such evidence when direct comparisons are limited, the strength of conclusions is necessarily tempered by reliance on the transitivity assumption and the observational nature of the underlying studies.

## Conflicts of Interest

No benefits in any form have been received or will be received related directly to this article.
