# Supplementary material for: Open Versus Arthroscopic Reduction for Perilunate and Lunate Dislocations: A Systematic Review and Network Meta-Analysis
Source: J Hand Surg Glob Online. 2026 Aug 7;8(6):101010. doi: 10.1016/j.jhsg.2026.101010 (PMC13476706; doi:10.1016/j.jhsg.2026.101010)
Supplement: Supplementary file 1 [file mmc1.docx]

**Supplementary Table S1:** Risk of Bias Assessment Using Newcastle-Ottawa Scale for Observational Studies.

| **Study Name** | **Selection (max 4★)** | **Comparability (max 2★)** | **Outcome (max 3★)** | **Total NOS Score** | **Risk of Bias Rating** |
| --- | --- | --- | --- | --- | --- |
| Rachunek-Medved et al. 2025 | ★★★☆ | ☆☆ | ★★☆ | 5/9 | Moderate |
| Liechti et al. 2025 | ★★★★ | ★★ | ☆☆☆ | 6/9 | **Low** |
| Pappa et al. 2024 | ★★★☆ | ☆☆ | ★★★ | 6/9 | **Low** |
| Yu et al. 2022 | ★★☆☆ | ☆☆ | ★★★ | 5/9 | Moderate |
| Colak et al. 2021 | ★★☆☆ | ☆☆ | ★★☆ | 4/9 | Moderate |
| Ozyurekoglu et al. 2021 | ★★☆☆ | ☆☆ | ★★☆ | 4/9 | Moderate |
| George et al. 2020 | ★★☆☆ | ☆☆ | ★★★ | 5/9 | Moderate |
| Kural et al. 2020 | ★★☆☆ | ☆☆ | ★★★ | 5/9 | Moderate |
| Dunn et al. 2018 | ★★★☆ | ☆☆ | ★★★ | 6/9 | **Low** |
| Meszaros et al. 2018 | ★★☆☆ | ☆☆ | ★★★ | 5/9 | Moderate |
| Oh et al. 2017 | ★★★★ | ★☆ | ★★★ | 7/9 | **Low** |
| Pinho et al. 2017 | ★☆☆☆ | ☆☆ | ☆☆☆ | 1/9 | **High** |
| Israel et al. 2016 | ★★★★ | ☆☆ | ★★★ | 7/9 | **Low** |
| Kim et al. 2015 | ★★☆☆ | ☆☆ | ★★★ | 5/9 | Moderate |
| Herzberg et al. 2015 | ★★☆☆ | ☆☆ | ★★☆ | 4/9 | Moderate |
| Kara et al. 2015 | ★★☆☆ | ☆☆ | ★★☆ | 4/9 | Moderate |
| Krief et al. 2015 | ★★★☆ | ☆☆ | ★★☆ | 5/9 | Moderate |
| Garg et al. 2012 | ★★☆☆ | ☆☆ | ★★★ | 5/9 | Moderate |
| Chou et al. 2012 | ★★☆☆ | ☆☆ | ★★☆ | 4/9 | Moderate |
| Kim et al. 2012 | ★★☆☆ | ☆☆ | ★★★ | 5/9 | Moderate |
| Malovic et al. 2011 | ★★★☆ | ☆☆ | ★★★ | 6/9 | **Low** |
| Dhillon et al. 2011 | ★★☆☆ | ☆☆ | ★★★ | 5/9 | Moderate |
| Kailu et al. 2010 | ★★☆☆ | ☆☆ | ★★☆ | 4/9 | Moderate |
| Forli et al. 2010 | ★★☆☆ | ☆☆ | ★★☆ | 4/9 | Moderate |
| Kremer et al. 2010 | ★★★☆ | ☆☆ | ★★☆ | 5/9 | Moderate |
| Wong et al. 2008 | ★★☆☆ | ☆☆ | ★★☆ | 4/9 | Moderate |
| Komurcu et al. 2008 | ★★☆☆ | ☆☆ | ★★★ | 5/9 | Moderate |
| Knoll et al. 2005 | ★★☆☆ | ☆☆ | ★★★ | 5/9 | Moderate |
| Trumble et al. 2004 | ★★☆☆ | ☆☆ | ★★★ | 5/9 | Moderate |
| Herzberg et al. 2002 | ★★☆☆ | ☆☆ | ★★☆ | 4/9 | Moderate |
| Hildebrand et al. 2000 | ★★☆☆ | ☆☆ | ★★★ | 5/9 | Moderate |
| Sotereanos et al. 1997 | ★★☆☆ | ☆☆ | ★★★ | 5/9 | Moderate |
| Herzberg et al. 1993 | ★★★★ | ☆☆ | ★★☆ | 6/9 | **Low** |
| Inoue et al. 1990 | ★★☆☆ | ☆☆ | ★☆☆ | 3/9 | **High** |

**Supplementary Table S2:** GRADE Evidence Profile For Quality Assessment for Open versus Arthroscopic Reduction of Perilunate/Lunate Dislocations.

| **Outcome** | **No. of Studies (Participants)** | **Study Design** | **Risk of Bias** | **Inconsistency** | **Indirectness** | **Imprecision** | **Publication Bias** | **Other Considerations** | **Quality Rating** | **Summary of Findings** |
| --- | --- | --- | --- | --- | --- | --- | --- | --- | --- | --- |
| **Mayo Wrist Score** (Functional Recovery) | 15 studies (438 participants) | 2 comparative studies, 13 case series | Serious¹ (-1) | Not serious | Not serious | Serious² (-1) | Undetected | None | ⊕⊝⊝⊝ **VERY LOW** | Arthroscopic reduction may improve functional scores (SMD 1.036, 95% CI 0.43-1.64), but evidence is very uncertain |
| **DASH Score** (Disability Assessment) | 5 studies (148 participants) | 1 comparative study, 4 case series | Serious¹ (-1) | Not serious | Not serious | Very serious³ (-2) | Undetected | Large effect⁴ (+1) | ⊕⊝⊝⊝ **VERY LOW** | Arthroscopic reduction may significantly reduce disability (SMD -1.97, 95% CI -3.04 to -0.90), but evidence is very uncertain |
| **Anatomical Success Rate** | 12 studies (300 participants) | 1 comparative study, 11 case series | Serious¹ (-1) | Not serious | Not serious | Serious⁵ (-1) | Undetected | None | ⊕⊝⊝⊝ **VERY LOW** | There may be little to no difference in anatomical success between approaches (RR 1.13, 95% CI 0.89-1.42) |
| **Overall Complication Rate** | 28 studies (678 participants) | 1 comparative study, 27 case series | Serious¹ (-1) | Serious⁶ (-1) | Not serious | Very serious⁷ (-2) | Suspected⁸ (-1) | None | ⊕⊝⊝⊝ **VERY LOW** | Arthroscopic reduction may reduce complications (RR 0.40, 95% CI 0.09-1.80), but evidence is very uncertain |
| **Nonunion Rate** | 22 studies (478 participants) | 1 comparative study, 21 case series | Serious¹ (-1) | Not serious | Not serious | Very serious⁹ (-2) | Undetected | None | ⊕⊝⊝⊝ **VERY LOW** | The effect on nonunion rates is very uncertain (RR 0.33, 95% CI 0.02-7.44) due to very few events |
| **Post-traumatic Arthritis** | 18 studies (420 participants) | 1 comparative study, 17 case series | Very serious¹⁰ (-2) | Very serious¹¹ (-2) | Serious¹² (-1) | Very serious¹³ (-2) | Strongly suspected¹⁴ (-1) | None | ⊕⊝⊝⊝ **VERY LOW** | The effect on arthritis development is very uncertain due to multiple serious limitations |
| **Grip Strength Recovery** | 16 studies (398 participants) | 2 comparative studies, 14 case series | Serious¹ (-1) | Very serious¹⁵ (-2) | Not serious | Serious¹⁶ (-1) | Undetected | None | ⊕⊝⊝⊝ **VERY LOW** | Arthroscopic reduction may improve grip strength (+8.2% vs contralateral), but evidence is very uncertain |

**Supplementary Table S3:** Statistical Tests and Sensitivity Analyses.

| **Outcome/Analysis** | **Heterogeneity Assessment** | **Subgroup Analysis** | **Sensitivity Testing** | **Adjustment Methods** |
| --- | --- | --- | --- | --- |
| **Mayo Wrist Score (Direct)** | **I² = 0.0%**, Q = 0.21 (p = 0.647), τ² = 0.000 | Acute: I² = 15.2%, Experienced surgeons: I² = 0.0% (**p = 0.045**) | Leave-one-out: Max Δ = 0.115 SMD, Fixed = Random effects | Random effects applied, Bonferroni α = 0.007 |
| **DASH Score (Direct)** | **Single study** - heterogeneity not assessable | Not applicable - insufficient data | Not applicable - single study | Complete case analysis |
| **Complications (Direct)** | **Single study** - heterogeneity not assessable | Fracture pattern: Greater arc 37.1% vs Lesser arc 16.5% (**p = 0.002**) | Not applicable - single study | Missing data exclusion applied |
| **Mayo Score (Open Pool)** | **I² = 50.8%**, Q = 28.45 (p = 0.012), τ² = 142.3 | Quality: High NOS I² = 18.7% vs Moderate I² = 31.2% (p = 0.156) | Quality restriction: Minimal impact on estimates | Random effects, quality weighting applied |
| **Mayo Score (Arthroscopy Pool)** | **I² = 59.5%**, Q = 12.34 (p = 0.030), τ² = 8.7 | Technique variation: I² = 22.4% vs 35.6% (p = 0.312) | Alternative measures: SMD vs MD consistent | Knapp-Hartung adjustment for small n |
| **Complications (Open Pool)** | **I² = 54.0%**, Q = 45.67 (p = 0.002), τ² = 0.087 | Sample size: Large (≥30) I² = 12.8% vs Small I² = 38.9% (**p = 0.089**) | Outlier detection: No extreme outliers identified | Random effects, missing data handling |
| **Complications (Arthroscopy Pool)** | **I² = 32.7%**, Q = 8.92 (p = 0.178), τ² = 0.024 | Consistent across subgroups: No significant interactions | Results stable across sensitivity scenarios | Standard random effects |
